# Advances in Anesthesia for Shoulder Surgery: A Comprehensive Review of Dexmedetomidine-Enhanced Interscalene Brachial Plexus Block

**DOI:** 10.7759/cureus.48827

**Published:** 2023-11-15

**Authors:** Varun N Thawkar, Karuna Taksande

**Affiliations:** 1 Anesthesiology, Acharya Vinoba Bhave Rural Hospital, Jawaharlal Nehru Medical College, Datta Meghe Institute of Higher Education & Research, Wardha, IND

**Keywords:** anxiolysis, pain management, regional anesthesia, shoulder surgery, interscalene block, dexmedetomidine

## Abstract

Surgical procedures on the shoulder pose distinctive challenges in managing pain during the perioperative period, underscoring the importance of exploring innovative anesthesia techniques. This comprehensive review article delves into integrating dexmedetomidine, an alpha-2 adrenergic agonist, within interscalene brachial plexus blocks for shoulder surgery. The review initiates by underscoring the pivotal role of effective anesthesia in shoulder surgery and elucidates the rationale behind investigating dexmedetomidine as an adjunct. It meticulously examines the anatomy and physiology of the brachial plexus, emphasizing its critical significance in shoulder surgery. Furthermore, the article expounds on dexmedetomidine's mechanisms of action and pharmacokinetics, encompassing its safety profile and potential side effects. The conventional interscalene brachial plexus block techniques, along with their limitations and challenges, are discussed, laying the foundation for the integration of dexmedetomidine. The review subsequently delves into exploring the role of dexmedetomidine in regional anesthesia, covering previous studies, mechanisms of action, and the potential advantages of incorporating it into nerve blocks. The review's core concentrates on the practical application of dexmedetomidine-enhanced interscalene brachial plexus blocks. This includes discussions on administration techniques, dosage guidelines, and compelling evidence supporting its utilization. Clinical scenarios where this approach proves most advantageous are thoroughly explored, comparing its effectiveness with traditional techniques in terms of pain control and patient outcomes. A comprehensive examination of relevant clinical trials and case studies highlights the evidence supporting its efficacy. The review also underscores safety considerations associated with dexmedetomidine. It proposes strategies for mitigating risks to ensure patient safety. Insights into future directions and research are provided, encompassing ongoing studies, areas necessitating further investigation, and potential refinements in technique. Finally, the article summarizes key findings, emphasizing the practicality of dexmedetomidine-enhanced interscalene brachial plexus blocks in shoulder surgery and its far-reaching implications for clinical practice and patient care.

## Introduction and background

Advancements in shoulder surgery have positioned it as a vital component of orthopedic practice, addressing a spectrum of conditions like rotator cuff tears, labral injuries, and osteoarthritis. The intricate mobility of the shoulder joint, integral to daily activities, underscores the impact of shoulder pathology on patients' quality of life, often necessitating surgical interventions for pain relief and functional restoration [1].

Orthopedic surgeons employ diverse techniques to address shoulder issues, including arthroscopy, open surgery, and joint replacement. While surgical proficiency is crucial for success, the choice of anesthetic management holds equal importance in ensuring patient comfort, optimizing surgical conditions, and minimizing postoperative pain. This review explores the evolving landscape of anesthesia in shoulder surgery, focusing on the use of dexmedetomidine-enhanced interscalene brachial plexus block-an emerging technique with the potential to revolutionize the perioperative experience for shoulder surgery patients [2,3].

Effective anesthesia is fundamental for safe and successful surgical procedures, particularly in shoulder surgery where intraoperative collaboration with the patient is often essential. Unlike many other orthopedic surgeries, shoulder procedures frequently require active patient participation to assess joint mobility, stability, and overall function. This poses unique challenges for anesthesia providers who must ensure pain control and patient comfort without compromising the patient's involvement in the surgical process [4-6].

Postoperative pain management is of paramount importance in shoulder surgery due to the intricate anatomy and rich innervation of the shoulder, which can lead to significant pain if not adequately addressed. In recent years, regional anesthesia techniques, particularly the interscalene brachial plexus block, have gained prominence for providing targeted analgesia to the shoulder and upper extremities while allowing patient consciousness and interaction with the surgeon during the procedure [4,7].

Dexmedetomidine, a highly selective alpha-2 adrenergic agonist, has emerged as a potential adjuvant to traditional interscalene brachial plexus block. Its sedative and analgesic properties, combined with a favorable side-effect profile, make it an attractive option for enhancing the quality and duration of analgesia provided by the block. Dexmedetomidine's ability to induce conscious sedation without respiratory depression is particularly valuable in maintaining intraoperative collaboration, a critical aspect of shoulder surgery [8].

This comprehensive review aims to examine the current state of dexmedetomidine-enhanced interscalene brachial plexus block in shoulder surgery. The exploration will cover anatomical and physiological considerations, mechanisms of action, administration techniques, clinical applications, safety profiles, and potential future directions in this evolving field. The review will logically progress, starting with the significance of shoulder surgery, the role of anesthesia, and the rationale for considering dexmedetomidine-enhanced interscalene brachial plexus block. It will then delve into various aspects of this technique, concluding with a comprehensive assessment of its potential to enhance the field of shoulder surgery anesthesia.

## Review

Anatomy and physiology

Overview of the Brachial Plexus

To comprehend the role of dexmedetomidine-enhanced interscalene brachial plexus block in shoulder surgery, it is imperative to grasp the anatomy and physiology of the brachial plexus. The brachial plexus is a complex network of nerves originating from the cervical and upper thoracic spinal nerve roots (C5-T1) and is responsible for innervating the upper extremities. This intricate neural structure is essential for motor and sensory function in the shoulder, arm, and hand [9]. The brachial plexus can be divided into five main roots or trunks (superior, middle, and inferior), giving rise to divisions, cords, and individual peripheral nerves. The interplay between these nerve structures provides the necessary neural input to control the shoulder and upper limb. Understanding the anatomical relationships within the brachial plexus is crucial for achieving effective regional anesthesia while minimizing potential complications [10].

Innervation of the Shoulder Region

Suprascapular nerve: The suprascapular nerve, originating from the superior trunk of the brachial plexus (composed of nerve roots C5-C6), plays a crucial role in the innervation of certain shoulder muscles. It provides motor innervation to the supraspinatus and infraspinatus muscles. The supraspinatus muscle contributes to shoulder abduction during the initial phase of this movement, while the infraspinatus muscle aids in the external rotation of the shoulder joint. These muscles are vital for shoulder stability and rotator cuff function [11].

Axillary nerve: The axillary nerve arises from the posterior cord of the brachial plexus (composed of nerve roots C5-C6). It primarily innervates the deltoid muscle, which is responsible for shoulder abduction. The deltoid muscle allows the arm to be lifted from the body's side. Damage to the axillary nerve can result in weakness or dysfunction of the deltoid muscle, leading to difficulty raising the arm [12].

Lateral and medial pectoral nerves: While not exclusively shoulder muscles, the pectoralis major muscles receive motor innervation from the lateral and medial pectoral nerves, respectively. These nerves originate from the lateral and medial cords of the brachial plexus. Although the primary function of the pectoralis major is related to the chest, these muscles also contribute to certain shoulder movements and stability. For example, they play a role in activities that involve adduction and internal rotation of the arm [13].

Long thoracic nerve: The long thoracic nerve originates from the nerve roots of C5-C7. It innervates the serratus anterior muscle. The serratus anterior muscle is crucial for maintaining the scapula (shoulder blade) against the thoracic wall. This function is vital for normal shoulder movement and stability, especially during overhead arm movement activities. Damage to the long thoracic nerve can result in a condition known as "winged scapula," which can impair shoulder function and stability [14].

Role of the Brachial Plexus in Shoulder Surgery

The brachial plexus plays a pivotal role in shoulder surgery, as it houses the nerves responsible for both motor and sensory functions in the shoulder and upper limb. During surgical procedures on the shoulder, such as rotator cuff repairs, labral repairs, or joint replacements, it is essential to provide effective anesthesia that renders the shoulder region insensate to pain. This ensures patient comfort and allows the surgeon to manipulate the joint and assess its function without causing distress to the patient [15].

The interscalene brachial plexus block has emerged as a favored technique for achieving regional anesthesia in shoulder surgery. By precisely targeting the brachial plexus at the level of the interscalene groove in the neck, this block can provide complete sensory blockade to the shoulder, arm, and hand. Importantly, the block's mechanism facilitates anesthesia from the distal portion of the limb to the clavicle and the midshaft of the humerus. Furthermore, motor function remains preserved, allowing patients to actively participate in the surgical process when required [16]. Understanding the anatomy and physiology of the brachial plexus is paramount when performing and enhancing interscalene blocks with adjuncts like dexmedetomidine. Dexmedetomidine's selective actions can further refine the precision and efficacy of this regional anesthesia technique, making it an exciting avenue for exploration in shoulder surgery.

Dexmedetomidine: mechanism of action and pharmacology

Dexmedetomidine

Dexmedetomidine is a relatively recent addition to the armamentarium of anesthetic agents, gaining recognition for its unique properties and versatility in clinical practice. It belongs to the class of alpha-2 adrenergic agonists, and its pharmacological characteristics make it well-suited for various applications, including sedation, analgesia, and adjunctive therapy in regional anesthesia [17].

Mechanism of Action of Dexmedetomidine

Sedation and anxiolysis: Dexmedetomidine's mechanism of action primarily involves its selective activation of alpha-2 adrenergic receptors in the central nervous system, particularly in the locus coeruleus. This activation reduces the release of norepinephrine, resulting in a calming and sedative effect. Patients who receive dexmedetomidine often experience reduced anxiety and increased cooperation, which can be particularly beneficial in regional anesthesia procedures where patient comfort and cooperation are essential. The sedative and anxiolytic properties of dexmedetomidine contribute to a more relaxed and less apprehensive patient experience during the procedure [18].

Analgesia: Dexmedetomidine, recognized for its analgesic properties, operates through a multifaceted mechanism to relieve pain. In the realm of regional anesthesia, it can augment the effectiveness of local anesthetics. Operating at the spinal cord level, dexmedetomidine modulates pain signaling pathways, thereby diminishing pain perception. This attribute is particularly advantageous in objectively extending the duration of analgesia facilitated by regional blocks. Numerous research studies have explored the potential of dexmedetomidine as a perineural adjunct, focusing on its impact on the mean/median duration of the prolongation of effect and the objective assessment of analgesic quality, often measured through pain scores. The findings of these investigations collectively contribute to a comprehensive understanding of the efficacy of dexmedetomidine in reducing the requirement for rescue analgesia in the postoperative period [19].

Sympatholysis: Dexmedetomidine exhibits a significant sympatholytic effect, attributed to the activation of alpha-2 receptors, resulting in reduced sympathetic outflow from the central nervous system. This action leads to a decrease in heart rate and blood pressure. While this reduction in sympathetic tone can offer advantages in specific patient populations, such as those with hypertension or cardiovascular comorbidities, it necessitates vigilant monitoring of hemodynamic parameters. Anesthesiologists and healthcare providers must be prepared to manage potential hemodynamic changes, including bradycardia and hypotension, especially during dexmedetomidine bolus administration or high-dose infusions [20].

Preservation of respiratory drive: Dexmedetomidine typically preserves respiratory drive, unlike some sedative agents. This property makes it a safer choice for patients requiring conscious sedation during procedures, as it reduces the risk of respiratory depression. This is particularly relevant in regional anesthesia, where maintaining spontaneous respiration is crucial, especially when patients must actively participate in the surgical process [21].

Pharmacokinetics and Pharmacodynamics

Dexmedetomidine is administered intravenously and exhibits a rapid onset of action, typically within minutes of administration. Its pharmacokinetic properties include a relatively short half-life of approximately 2 hours, facilitating precise control over sedation levels and allowing for rapid recovery once the infusion is discontinued [22]. Pharmacodynamically, dexmedetomidine offers a titratable sedative effect, allowing healthcare providers to adjust the depth of sedation to match the patient's needs, from light sedation to deep sedation or even a state of near-general anesthesia [23].

Safety Profile and Side Effects

Hypotension and bradycardia: Dexmedetomidine's primary mechanism of action involves its selective activation of alpha-2 adrenergic receptors in the central nervous system, which leads to reduced sympathetic outflow. While this property contributes to its analgesic and anxiolytic effects, it can also significantly decrease blood pressure and heart rate, particularly when dexmedetomidine is administered as a bolus or at higher infusion rates [17]. Maintaining hemodynamic stability during dexmedetomidine-enhanced blocks is essential. Anesthesiologists and healthcare providers must closely monitor blood pressure and heart rate, especially in patients with preexisting cardiovascular conditions or those more susceptible to bradycardia and hypotension. Prompt interventions, such as dose adjustments or the administration of vasoactive medications, may be necessary to manage these hemodynamic effects [24].

Sedation-related issues: Dexmedetomidine's sedative and anxiolytic properties, while advantageous in promoting patient comfort and cooperation, can also pose risks. Excessive sedation, especially when dexmedetomidine is administered at high doses or rapidly, can lead to respiratory depression. This concerns patients with compromised respiratory function, such as those with chronic obstructive pulmonary disease (COPD) or sleep apnea [17]. Careful titration of dexmedetomidine dosing is essential to achieve the desired level of sedation and analgesia while avoiding excessive sedation and its associated respiratory complications. Monitoring respiratory parameters, including oxygen saturation and end-tidal carbon dioxide levels, is critical to detect any signs of respiratory depression promptly [17].

Nausea and vomiting: Nausea and vomiting are potential side effects of dexmedetomidine administration. Some patients may experience these symptoms during or after the procedure. Nausea and vomiting can be uncomfortable for patients and may necessitate the administration of antiemetic medications to alleviate these side effects [25]. The risk of nausea and vomiting should be considered when planning the anesthesia regimen, and antiemetic prophylaxis may be administered as a preventive measure.

Delayed recovery: Dexmedetomidine has a relatively short half-life compared to other sedative agents, which generally facilitates a faster recovery. However, in some cases, prolonged sedation may occur, resulting in a longer time to complete recovery. This can impact the patient's ability to mobilize and achieve discharge criteria, potentially affecting the overall length of the hospital stay or time spent in the recovery area [20]. Healthcare providers should be prepared for potential delays in recovery and plan postoperative care accordingly, ensuring that patients are closely monitored until they are fully awake and alert.

Interscalene brachial plexus block

Interscalene Brachial Plexus Block

The interscalene brachial plexus block is a well-established regional analgesia and shoulder surgery technique. It involves the precise placement of local anesthetics near the brachial plexus as it passes through the interscalene groove between the neck's anterior and middle scalene muscles. This block is favored for shoulder surgery because it can achieve targeted shoulder, arm, and hand anesthesia while preserving motor function [26]. The block is typically performed using ultrasound guidance or nerve stimulation techniques to identify the brachial plexus's location accurately. Once the target is identified, a local anesthetic solution is injected to bathe the nerves, resulting in a reversible blockade of sensory and motor function [27].

Traditional Techniques and Agents Used

Traditionally, the interscalene brachial plexus block has been accomplished using local anesthetics, such as bupivacaine or ropivacaine. These agents provide effective anesthesia by blocking nerve conduction in the brachial plexus, resulting in sensory loss and muscle relaxation. Opioids, such as fentanyl or morphine, are sometimes added to the local anesthetic mixture to enhance postoperative pain relief [28]. Techniques for administering the block have evolved, with the advent of ultrasound guidance offering improved precision and safety. Ultrasound allows for real-time visualization of nerve structures, reducing the risk of accidental intravascular injection and increasing the likelihood of successful nerve blockade. Nerve stimulation techniques, which involve using a stimulating needle to elicit muscle twitches, have also been employed to confirm needle placement within proximity to the nerves [29].

Limitations and Challenges of Traditional Approaches

Motor function preservation: One of the primary challenges with traditional approaches to interscalene brachial plexus blocks is achieving the delicate balance between sensory anesthesia and motor function preservation. While the primary goal is to provide effective pain relief, inadvertent motor blockade can occur, resulting in motor weakness. This limitation can be especially problematic for procedures requiring patient cooperation and active participation, such as certain types of shoulder surgery [30]. Surgeons often rely on the patient's ability to move the affected arm during the procedure, and excessive motor blockade can hinder this cooperation. Striking the right balance between adequate analgesia and motor preservation is a clinical challenge that anesthesiologists must navigate during traditional block techniques [31].

Duration of analgesia: Traditional local anesthetics used in interscalene brachial plexus blocks may provide adequate analgesia during the surgery, but their duration of action can be limited. Patients frequently experience a decrease in the effectiveness of the block in the hours following surgery. As a result, many patients require additional analgesia, often in the form of systemic opioids, to manage postoperative pain [32]. The reliance on systemic opioids for pain control in the postoperative period introduces the risk of opioid-related side effects, including respiratory depression, nausea, and constipation. Additionally, opioid use can delay recovery and increase the length of hospital stays, which may not align with modern fast-track surgery protocols [33].

Hemodynamic effects: Traditional approaches to interscalene brachial plexus blocks involve the administration of a relatively high volume of local anesthetic in the interscalene groove. This can lead to systemic absorption of the local anesthetic, which, in turn, can result in transient hemodynamic changes, including hypotension. Hypotension can be of particular concern in patients with preexisting cardiovascular conditions or hemodynamically unstable patients [34]. Managing hemodynamic fluctuations during the procedure can be challenging and may require interventions such as intravenous fluid administration or vasopressor support. These interventions can complicate anesthesia management and may not be ideal for certain patient populations [35].

Risk of systemic toxicity: One of the most significant concerns with traditional interscalene brachial plexus blocks is the risk of local anesthetic systemic toxicity (LAST). LAST can occur when high doses of local anesthetic are administered or when the local anesthetic is rapidly absorbed into the systemic circulation [36]. LAST can manifest as central nervous system and cardiovascular toxicity, leading to symptoms such as seizures, cardiac arrhythmias, and even cardiac arrest. Recognizing and managing LAST requires prompt and skilled intervention, and the risk is higher when traditional techniques involve large volumes of local anesthetic [37].

Dexmedetomidine in regional anesthesia

Dexmedetomidine in Regional Anesthesia

Prolonged analgesia: Multiple studies have consistently demonstrated that adding dexmedetomidine to local nerve block anesthetics significantly prolongs analgesia duration. This effect is particularly notable in peripheral nerve blocks, where dexmedetomidine enhances the action of the local anesthetic agent. This prolongation's precise mechanism involves dexmedetomidine's local and systemic effects [38]. Dexmedetomidine's local action at the site of the nerve block, including its presynaptic inhibition and modulation of nociceptive signaling, contributes to the extended duration of pain relief. This extended analgesic effect is paramount in regional anesthesia, as it helps ensure that patients remain comfortable during and after surgical procedures, reducing the need for additional analgesic interventions [38].

Reduced local anesthetic volume: The use of dexmedetomidine as an adjunct to local anesthetics allows for a reduction in the volume of local anesthetic required to achieve effective anesthesia. This reduction in local anesthetic volume is advantageous for several reasons; one is mitigating the risk of local anesthetic systemic toxicity (LAST) [39]. Minimizing the amount of local anesthetic administered significantly reduces the likelihood of systemic absorption and toxic effects. This is particularly critical in nerve blocks where precise dosing and minimizing systemic exposure are key considerations for patient safety. Dexmedetomidine's ability to enhance the local anesthetic's action allows for more sparing use of these agents, further contributing to patient safety [36].

Improved postoperative pain control: Dexmedetomidine has consistently shown promise in improving postoperative pain control when used as an adjuvant in regional anesthesia. Patients who receive dexmedetomidine-enhanced blocks frequently report lower pain scores in the immediate postoperative period, translating to enhanced postoperative comfort [40]. Additionally, reduced postoperative pain leads to a decreased need for systemic opioids, which is highly relevant in the opioid epidemic and concerns surrounding opioid-related side effects and complications. Dexmedetomidine's opioid-sparing effect is particularly beneficial for patients undergoing regional anesthesia, as it aligns to minimize opioid use [41].

Sedative and anxiolytic effects: Dexmedetomidine's sedative and anxiolytic properties are valuable in the context of regional anesthesia procedures. Patients often experience reduced anxiety and a calmer perioperative experience when dexmedetomidine is included in their anesthesia regimen. This is especially relevant in procedures where patients remain conscious and cooperative during surgery, such as interscalene brachial plexus blocks for shoulder surgery [42]. Dexmedetomidine enhances patient comfort and cooperation during the procedure by promoting calm and relaxation without inducing deep unconsciousness. Patients can better tolerate the surgical experience, leading to improved surgical outcomes and a more positive overall experience [43].

Hemodynamic stability: Dexmedetomidine's sympatholytic effects are particularly important in patients with cardiovascular comorbidities undergoing regional anesthesia. These effects contribute to hemodynamic stability during procedures, helping to maintain normal heart rate and blood pressure [44]. Patients with preexisting cardiovascular conditions may be at increased risk of hemodynamic fluctuations during surgery. Dexmedetomidine's ability to reduce sympathetic tone helps mitigate these risks, ensuring patients remain stable throughout the procedure. This contributes to overall patient safety and enhances the feasibility of regional anesthesia in high-risk populations [44].

Mechanisms of Dexmedetomidine's Action in Regional Anesthesia

Presynaptic inhibition: Dexmedetomidine's primary mechanism of action in regional anesthesia involves presynaptic inhibition. When administered locally, dexmedetomidine acts on presynaptic nerve endings by binding to alpha-2 adrenergic receptors. This binding inhibits the release of norepinephrine, a neurotransmitter responsible for transmitting pain signals along the nerves [21]. By reducing the release of norepinephrine, dexmedetomidine effectively decreases sympathetic outflow in the targeted area. This reduction in sympathetic activity has a dual benefit in the context of interscalene brachial plexus blocks. First, it minimizes sympathetic-mediated vasoconstriction, promoting vasodilation and enhancing local blood flow around the nerves. Improved blood flow helps ensure the efficient distribution of local anesthetic and dexmedetomidine to the nerve fibers, enhancing the block's efficacy. Second, it dampens the transmission of nociceptive signals, resulting in profound analgesia within the surgical area. Patients experience reduced pain perception, contributing significantly to their comfort during the procedure and in the immediate postoperative period [45].

Peripheral actions: Dexmedetomidine's direct actions on peripheral nerves are another important aspect of its mechanism in regional anesthesia. At the site of the nerve block, dexmedetomidine interacts with nerve endings, modulating nociceptive signaling locally. This direct effect is particularly relevant in enhancing the precision and efficacy of the block [46]. Dexmedetomidine's presence at the peripheral nerve endings helps fine-tune the pain transmission blockade. It contributes to a more targeted and specific blockade of pain signals originating from the shoulder region. By directly influencing the peripheral nerves, dexmedetomidine ensures that pain relief is optimized at the surgical site, minimizing the potential for breakthrough pain and enhancing patient comfort [9].

Alpha-2 agonist effects: Dexmedetomidine's selective activation of alpha-2 adrenergic receptors in the central nervous system plays a pivotal role in its mechanism of action during regional anesthesia. These effects are particularly beneficial for patient comfort and cooperation during surgical procedures [47].

Sedation: Dexmedetomidine's sedative properties promote a state of calm and relaxation without inducing deep unconsciousness. Patients often remain conscious and responsive but experience reduced anxiety and discomfort. This level of sedation ensures patients are comfortable and cooperative during the regional anesthesia procedure. This is especially relevant for procedures like interscalene brachial plexus blocks where patient interaction may be required [48].

Anxiolysis: Dexmedetomidine's anxiolytic effects are crucial for alleviating anxiety and apprehension in patients undergoing regional anesthesia. These effects contribute to a more pleasant perioperative experience and help reduce the psychological stress associated with surgery [49].

Reduced sympathetic tone: The activation of alpha-2 adrenergic receptors by dexmedetomidine leads to a decrease in sympathetic tone, resulting in lowered heart rate and blood pressure. This hemodynamic stability is valuable during regional anesthesia procedures, as it minimizes the risk of hemodynamic fluctuations and ensures patient safety [17].

Benefits and Potential Advantages of Using Dexmedetomidine in Nerve Blocks

Prolonged analgesia: One of the primary benefits of incorporating dexmedetomidine into nerve blocks is its ability to extend the analgesia duration significantly. When administered locally, dexmedetomidine acts directly on adrenergic receptors in nerve endings, leading to prolonged pain relief in the targeted area. This prolonged analgesic effect is particularly advantageous for postoperative pain management as it reduces the need for frequent reinjections of local anesthetics or systemic opioids [46]. In interscalene brachial plexus blocks for shoulder surgery, patients can experience extended pain relief during the critical early postoperative period, enhancing their overall comfort and facilitating a smoother recovery process [50].

Reduced local anesthetic requirements: Dexmedetomidine's ability to enhance the action of local anesthetics has the added benefit of allowing for the use of smaller volumes of these agents. By using lower volumes of local anesthetics, there is a reduced risk of local anesthetic systemic toxicity (LAST), a potentially severe complication associated with nerve blocks [32]. Reducing local anesthetic requirements improves the procedure's safety while maintaining effective pain control. It aligns with the principles of minimizing the total drug load while optimizing analgesia, which is especially important for patients with comorbidities or those who may be at higher risk of complications [51].

Improved patient comfort: Dexmedetomidine's sedative and anxiolytic effects are crucial in promoting improved patient comfort during regional anesthesia procedures. Patients undergoing shoulder surgery can experience significant anxiety and discomfort, especially when fully awake during the procedure. Dexmedetomidine's sedative properties can alleviate anxiety and provide a calming effect, making the perioperative experience more pleasant [20]. Additionally, the ability of dexmedetomidine to induce cooperative sedation allows patients to remain alert and responsive when needed, such as during neurologic assessments or when instructed by the surgical team. This balanced sedation improves patient cooperation and overall satisfaction [20].

Minimized opioid consumption: Dexmedetomidine's opioid-sparing effect is another notable advantage. By reducing the need for systemic opioids, patients are less likely to experience opioid-related side effects, including respiratory depression, nausea, vomiting, and constipation. This is particularly important in shoulder surgery, where the respiratory status and comfort of the patient are critical postoperatively [52]. Minimizing opioid use aligns with current trends in multimodal analgesia, promoting a more comprehensive and holistic approach to pain management while minimizing the risks associated with opioid medications [53].

Dexmedetomidine-enhanced interscalene brachial plexus block

Techniques for Administering Dexmedetomidine with Interscalene Brachial Plexus Block

Co-administration with local anesthetic: Co-administering dexmedetomidine with the local anesthetic mixture is a well-established technique for enhancing interscalene brachial plexus blocks. Dexmedetomidine is added to the local anesthetic solution, delivering both agents directly to the target nerves during the block procedure. This approach offers several advantages [39]. Combining dexmedetomidine with the local anesthetic ensures that dexmedetomidine's sedative and analgesic effects are localized to the surgical area, enhancing both the onset and duration of the block. This contributes to efficient pain relief and patient comfort during the procedure. Co-administration allows for a synergistic effect, where the local anesthetic provides sensory blockade while dexmedetomidine offers profound analgesia and sedation, improving the overall quality of regional anesthesia [17].

Bolus or infusion: The choice between administering dexmedetomidine as a bolus dose or as an infusion during the interscalene block depends on specific clinical considerations and surgical requirements [54].

Bolus administration: Dexmedetomidine can be given as a bolus dose for rapid onset of its effects. This is particularly beneficial when immediate sedation and analgesia are needed, such as during the initial stages of the procedure. The bolus dose is typically administered as a single injection, allowing swift action [20].

Infusion: Alternatively, dexmedetomidine can be administered as an infusion during the block procedure. This approach involves delivering dexmedetomidine gradually and continuously over time. Infusions are especially useful for longer-duration surgeries, providing a sustained effect. The infusion rate can be adjusted to achieve and maintain the desired level of sedation and analgesia throughout the procedure [23].

Single or multiple injection points: Deciding whether to inject dexmedetomidine at a single point or multiple points along the brachial plexus is contingent on the complexity of the surgery and the necessity for a comprehensive blockade [55].

Single injection point: In certain cases, a single injection point may suffice to achieve the required sensory blockade for the surgical site. This approach simplifies the procedure by minimizing the number of needle insertions. It is often suitable for less complex shoulder surgeries where a targeted blockade can adequately control pain [6].

Multiple injection points: For more extensive surgeries or when a broader blockade is essential, multiple injection points along the brachial plexus may be used. Distributing dexmedetomidine and the local anesthetic across multiple sites allows for a more comprehensive and widespread block. This is particularly advantageous for surgeries that involve multiple nerves and require extensive pain control [39].

Ultrasound guidance: Incorporating ultrasound guidance is indispensable when administering dexmedetomidine-enhanced interscalene brachial plexus blocks. Ultrasound technology provides real-time visualization of the targeted area's nerves, blood vessels, and surrounding structures [56]. Utilizing ultrasound ensures the accurate placement of the needle and the precise delivery of dexmedetomidine to the intended nerves. Real-time visualization allows healthcare providers to confirm the local anesthetic's spread and dexmedetomidine's spread, ensuring that the desired nerves are effectively blocked while minimizing the risk of complications [57]. Ultrasound guidance enhances safety by reducing the likelihood of accidental vascular puncture or nerve injury, allowing optimal control and precision during the block procedure. Clinicians must maintain proficiency in ultrasound-guided techniques to ensure accuracy and patient safety [58].

Dosage and Administration Guidelines

Dexmedetomidine concentration: Dexmedetomidine is available in various concentrations, typically ranging from 0.1 to 1.0 mcg/mL. The choice of dexmedetomidine concentration is a critical consideration in administering interscalene blocks. The selected concentration can significantly impact the total volume of solution administered and influence the onset and duration of the block [23]. Healthcare providers must carefully select the dexmedetomidine concentration that aligns with the specific requirements of the procedure and the patient's characteristics. This decision should be guided by the desired onset and duration of the block and considerations for patient safety [59].

Total dexmedetomidine dose: The dose of dexmedetomidine administered for interscalene blocks typically falls within a range of 0.25 to 1.0 mcg/kg. The specific dose chosen depends on several factors, including the patient's age, weight, and the anticipated duration of the surgical procedure [60]. Higher doses of dexmedetomidine are generally associated with longer durations of action, which can be advantageous for procedures with extended surgical times. However, it is crucial to exercise caution when administering higher doses, as individual patient factors, such as comorbidities and baseline hemodynamic stability, must be considered to avoid hemodynamic instability [60]. The precise dose should be determined through a comprehensive assessment of the patient's characteristics and the surgical context to achieve optimal sedation and analgesia while ensuring patient safety [60].

Rate of infusion: When dexmedetomidine is administered as an infusion, the typical infusion rate ranges from 0.2 to 0.7 mcg/kg/hour. The infusion rate is a critical parameter that requires careful adjustment to achieve the desired level of sedation and analgesia. Continuous monitoring of the patient's sedation level, vital signs, and response to the infusion is essential for effective titration. The infusion rate can be increased or decreased as needed to maintain the desired depth of sedation while avoiding excessive sedation or adverse effects such as hemodynamic instability. Healthcare providers should be attentive to changes in patient status and adapt the infusion rate accordingly to ensure that the patient remains comfortable and cooperative throughout the procedure [23].

Local anesthetic volume: An important safety consideration in dexmedetomidine-enhanced interscalene blocks is the volume of local anesthetic used in combination with dexmedetomidine. To minimize the risk of systemic toxicity, the volume of local anesthetic should be reduced compared to traditional techniques. The specific reduction in local anesthetic volume should be determined based on the type and concentration of the local anesthetic chosen. By using smaller volumes, clinicians can achieve the desired sensory blockade while minimizing the risk of systemic absorption and toxicity. Ensuring that the volume of local anesthetic remains within safe limits is a fundamental aspect of patient safety in dexmedetomidine-enhanced interscalene brachial plexus blocks, and adherence to established guidelines and best practices is paramount [46].

Evidence Supporting the Use of Dexmedetomidine

Prolonged analgesia: Numerous studies have provided strong evidence that dexmedetomidine enhances the duration of analgesia provided by interscalene brachial plexus blocks. Dexmedetomidine's alpha-2 adrenergic agonist properties extend the effect of local anesthetics, resulting in longer-lasting pain relief compared to traditional blocks using local anesthetics alone. This extended analgesia is especially valuable in shoulder surgery, where postoperative pain can be significant [45].

Improved postoperative pain control: Clinical trials consistently demonstrate that patients who receive dexmedetomidine-enhanced blocks report improved postoperative pain control. These patients experience reduced pain intensity, translating to decreased opioid consumption in the early postoperative period. This effect contributes to a more comfortable patient recovery experience and reduces the reliance on opioids, thereby minimizing opioid-related side effects [61].

Enhanced patient comfort: Dexmedetomidine's sedative and anxiolytic effects are well-documented and significantly enhance patient comfort during shoulder surgery. Patients receiving dexmedetomidine often report feeling calmer and less anxious, which fosters a cooperative and positive surgical environment. This is particularly important in shoulder surgery, where patient cooperation may be required for intraoperative adjustments and feedback [62].

Reduced opioid use and side effects: The opioid-sparing effect of dexmedetomidine is a key advantage. Studies consistently show reduced opioid requirements in patients who receive dexmedetomidine-enhanced blocks. Reducing opioid use helps mitigate opioid-related side effects, such as respiratory depression, nausea, and constipation. By minimizing these complications, dexmedetomidine contributes to a safer and more comfortable postoperative course [52].

Potential for same-day discharge: Dexmedetomidine-enhanced blocks have the potential to enable same-day discharge for select patients undergoing shoulder surgery. This is due to extended analgesia, reduced opioid requirements, and the absence of prolonged sedation or motor impairment. Same-day discharge protocols can be particularly advantageous for patients and healthcare systems by reducing hospital stays and associated costs while allowing patients to recover in their homes [21].

Clinical applications and efficacy

The Clinical Scenarios Where Dexmedetomidine-Enhanced Interscalene Brachial Plexus Block is Most Beneficial

Shoulder arthroscopy: Dexmedetomidine-enhanced blocks benefit patients undergoing shoulder arthroscopy procedures, such as rotator cuff or labral repairs. These minimally invasive surgeries often require precise patient cooperation, as intraoperative adjustments and feedback are essential. Dexmedetomidine provides profound analgesia while preserving motor function, enabling patients to participate in the surgical process actively. This enhances patient comfort and ensures optimal cooperation during the procedure, contributing to its success [63].

Joint replacement: Patients undergoing shoulder replacement surgeries, which may be more extensive and associated with significant postoperative pain, can benefit significantly from dexmedetomidine-enhanced blocks. Dexmedetomidine's ability to provide extended and robust pain relief in the early postoperative period is particularly valuable. It may expedite the rehabilitation process by allowing patients to engage in early mobilization and physical therapy, ultimately leading to improved shoulder function and faster recovery [64].

Same-day surgery: Dexmedetomidine is a valuable option for same-day discharge protocols, where patients undergo shoulder surgery and are discharged on the same day. Its potential to provide effective analgesia and sedation without the need for prolonged hospital stays contributes to the cost-effectiveness of these procedures. Patients can recover comfortably at home while benefiting from the extended analgesic effects of dexmedetomidine, promoting patient satisfaction and healthcare resource optimization [20].

High-risk patients: Patients with preexisting cardiovascular conditions or those at risk for opioid-related complications, such as respiratory depression, may particularly benefit from dexmedetomidine-enhanced blocks. Dexmedetomidine's opioid-sparing effect reduces the need for systemic opioids, minimizing the risks associated with opioid use in these high-risk populations. This approach aligns to enhance patient safety and optimize outcomes in vulnerable patient groups [65].

Patient preferences: Patient preferences are significant in anesthesia decisions. Some individuals may prefer regional anesthesia techniques incorporating dexmedetomidine due to the associated anxiolysis and perceived comfort during the procedure. The calming and sedative effects of dexmedetomidine can alleviate patient anxiety and contribute to a positive surgical experience [20].

Comparative Analysis with Traditional Techniques in Terms of Pain Control and Patient Outcomes

Pain control: Dexmedetomidine-enhanced blocks consistently demonstrate superior pain control compared to traditional blocks using local anesthetics alone. Dexmedetomidine's alpha-2 adrenergic agonist properties extend the duration and intensity of analgesia, leading to longer-lasting and more robust pain relief. Patients who receive dexmedetomidine-enhanced blocks often report reduced postoperative pain levels, translating to decreased opioid requirements and improved comfort during the early recovery phase [66].

Motor function preservation: One of the notable advantages of dexmedetomidine is its ability to preserve motor function. Patients undergoing shoulder surgery frequently require intact motor function for various reasons, such as intraoperative positioning adjustments or active participation in physical therapy. Dexmedetomidine allows for precise sensory blockade while maintaining motor function, enabling patients to cooperate with surgeons and rehabilitation teams. In contrast, traditional blocks may result in varying degrees of motor weakness, potentially limiting patients' ability to actively engage in their care [67].

Patient satisfaction: Dexmedetomidine's sedative and anxiolytic effects significantly increase patient satisfaction during the procedure. These properties help reduce patient anxiety and apprehension, promoting a more comfortable and cooperative surgical environment. Patients who receive dexmedetomidine-enhanced blocks often report feeling calmer and more at ease during surgery, which can positively impact their overall perception of the surgical experience [68].

Early rehabilitation: Enhanced pain control and preserved motor function provided by dexmedetomidine may facilitate earlier postoperative rehabilitation. Patients are more likely to engage in early mobilization and physical therapy when they experience less pain and maintain motor function. This can lead to improved shoulder function, quicker recovery, and potentially shorter hospital stays or outpatient recovery periods [69].

Reduced opioid use: Dexmedetomidine's opioid-sparing effect is a significant benefit, especially in the context of shoulder surgery. Patients receiving dexmedetomidine-enhanced blocks typically require fewer opioids for pain management, reducing the risk of opioid-related side effects such as respiratory depression, nausea, and constipation. This can lead to a more comfortable postoperative course and minimize the need for opioid reversal agents or interventions [70].

Clinical Trials and Case Studies

Extended duration of analgesia: Clinical trials consistently demonstrate that the supplementation of dexmedetomidine in regional anesthesia prolongs the duration of analgesia when compared to traditional blocks. This extended pain relief is especially evident in the studies of shoulder arthroscopy and joint replacement procedures. Dexmedetomidine's ability to enhance the duration of pain control provides patients with prolonged relief in the critical immediate postoperative period, reducing discomfort and improving overall surgical experience [71].

Improved pain scores: Patients receiving dexmedetomidine-enhanced blocks frequently report lower pain scores in the immediate postoperative period. These improved pain scores not only reflect enhanced comfort for patients but also signify the efficacy of dexmedetomidine in providing effective analgesia. Reduced pain intensity in the early postoperative phase contributes to a smoother recovery process and improved patient satisfaction [19].

Reduced opioid consumption: Clinical trials consistently reveal reduced opioid consumption in patients receiving dexmedetomidine-enhanced blocks. This reduction in opioid requirements indicates the potential of dexmedetomidine to mitigate opioid-related side effects and complications. By minimizing opioid use, patients experience fewer adverse effects, such as nausea, vomiting, and respiratory depression, contributing to safer and more comfortable postoperative care [72].

Patient satisfaction: Case studies often highlight individual patient experiences that underscore the advantages of dexmedetomidine in regional anesthesia for shoulder surgery. These case reports showcase reduced anxiety, enhanced patient cooperation during the procedure, and overall patient satisfaction. Dexmedetomidine's anxiolytic properties and ability to provide a calm and cooperative surgical environment contribute to a positive patient experience [73].

Safety profile: An essential aspect of clinical trials and case studies is the evaluation of safety parameters associated with dexmedetomidine administration. This includes monitoring hemodynamic stability and assessing the occurrence of adverse events. Dexmedetomidine's safety profile is generally favorable when administered judiciously and closely monitored. Clinical studies provide valuable insights into the balance between achieving the desired sedation and analgesia and maintaining hemodynamic stability, helping healthcare providers make informed decisions about its use [74].

Safety and side effects

Potential Side Effects and Complications Associated with Dexmedetomidine-Enhanced Blocks

Hemodynamic instability: Dexmedetomidine's alpha-2 adrenergic agonist properties can lead to hemodynamic instability, particularly during bolus administration or high-dose infusions. These effects may manifest as bradycardia (slow heart rate) and hypotension (low blood pressure). While these effects are generally well-tolerated and manageable, they can be of concern in patients with preexisting cardiovascular conditions, such as coronary artery disease or heart block. Careful dosing and continuous monitoring are essential to promptly address hemodynamic changes [75].

Sedation and respiratory depression: Dexmedetomidine is renowned for providing sedation without causing respiratory depression, making it a valuable choice for anesthesia. However, high doses or rapid dexmedetomidine infusions can result in excessive sedation, potentially leading to respiratory depression. This is particularly relevant in patients with compromised respiratory function, such as chronic obstructive pulmonary disease (COPD) or obstructive sleep apnea. Close monitoring of sedation levels and respiratory parameters is crucial to prevent and manage these effects [76].

Delayed recovery: Prolonged sedation and residual effects of dexmedetomidine may lead to delayed recovery, affecting the patient's ability to mobilize and discharge from the healthcare facility promptly. While the extended analgesic effects of dexmedetomidine can be advantageous in pain management, careful planning and monitoring are necessary to ensure that patients are sufficiently alert and responsive for safe postoperative recovery [76].

Local anesthetic toxicity: Co-administration of dexmedetomidine with local anesthetics may increase the total dose of medication delivered to the patient. This raises the risk of local anesthetic systemic toxicity (LAST), a rare but serious complication. LAST can manifest as neurologic symptoms, cardiovascular disturbances, or seizures and requires immediate treatment. Anesthesia providers should exercise caution and adhere to safe dosing practices to minimize the risk of LAST [36].

Nausea and vomiting: Some patients may experience nausea and vomiting as a side effect of dexmedetomidine. While these symptoms are generally mild, they can be uncomfortable and may necessitate additional antiemetic medications. Preventive measures, such as administering prophylactic antiemetics, can help reduce the incidence of postoperative nausea and vomiting [77].

Over-sedation: Dexmedetomidine's sedative properties, while desirable for providing patient comfort, can lead to excessive sedation if administered inappropriately or if the patient is particularly sensitive to its effects. Careful titration and monitoring are essential to maintain the desired level of sedation while avoiding over-sedation [78].

Allergic reactions: Although rare, allergic reactions to dexmedetomidine or other medication components can occur. These reactions may manifest as skin rashes, itching, swelling, or, in severe cases, anaphylaxis. Immediate recognition and prompt treatment of allergic reactions are critical to ensure patient safety [79].

Strategies for Mitigating Risks and Ensuring Patient Safety

Individualized patient assessment: A thorough preoperative assessment is critical to identify patient-specific factors that may influence the use of dexmedetomidine. This assessment should encompass the patient's medical history, comorbidities, medication regimen, and allergies or sensitivities. Identifying high-risk patients, such as those with cardiovascular disease or respiratory conditions, is essential to tailor the anesthesia plan accordingly [80].

Dosing considerations: Dexmedetomidine dosing should be customized based on the patient's characteristics and the surgical context. Patients at higher risk for hemodynamic instability may benefit from lower initial bolus doses and slower infusion rates. Careful consideration of the appropriate dosage ensures that the desired level of sedation and analgesia is achieved without excessive effects [23].

Hemodynamic monitoring: Continuous blood pressure and heart rate monitoring is crucial throughout the procedure, especially during dexmedetomidine administration. Monitoring allows for the early detection of hemodynamic changes and provides an opportunity for timely intervention. Healthcare providers should be prepared to manage potential episodes of bradycardia or hypotension [81].

Respiratory monitoring: Respiratory parameters, including oxygen saturation, end-tidal carbon dioxide (ETCO2), and respiratory rate, should be closely monitored to detect signs of respiratory depression. This is particularly important in patients with preexisting respiratory conditions or those receiving high doses of dexmedetomidine. Prompt recognition of respiratory issues is essential for patient safety [82].

Local anesthetic dosing: When dexmedetomidine is combined with local anesthetics, the volume of local anesthetic should be carefully considered to minimize the risk of local anesthetic systemic toxicity (LAST). Reducing the volume while maintaining effective analgesia can help mitigate the risk of this potential complication [83].

Careful titration: Dexmedetomidine dosing should be titrated carefully to achieve the desired level of sedation and analgesia while avoiding excessive sedation. Close monitoring of the patient's sedation level, responsiveness, and vital signs is essential to strike the right balance [84].

Antiemetic prophylaxis: Prophylactic administration of antiemetic medications can help reduce the risk of postoperative nausea and vomiting, which may occur as a side effect of dexmedetomidine. This can enhance patient comfort and satisfaction during recovery [85].

Patient education: Patients should be informed about the potential side effects of dexmedetomidine and the importance of close monitoring during the procedure. Open communication between patients and healthcare providers ensures that concerns or discomfort are promptly addressed [86].

Reversal agents: Anesthesia providers should be prepared to administer alpha-2 adrenergic receptor antagonists, such as atipamezole if excessive sedation or hemodynamic instability occurs. Having reversal agents readily available can help rapidly reverse the effects of dexmedetomidine if needed [87].

Continuous monitoring post-procedure: Monitoring of patients should continue into the postoperative period to ensure that they are recovering appropriately. Vital signs and comfort levels should be closely observed, and any signs of complications should be addressed promptly [88].

Future directions and research

Ongoing Research and Emerging Trends in the Use of Dexmedetomidine in Anesthesia for Shoulder Surgery

Expanded applications: Research explores the expanded applications of dexmedetomidine-enhanced blocks in a broader range of shoulder surgeries. This includes procedures beyond the typical scope of shoulder arthroscopy and joint replacement, such as revisions, complex reconstructions, and arthroplasties. Investigating the feasibility and benefits of dexmedetomidine in these diverse surgical scenarios will provide insights into its versatility and potential to enhance patient outcomes in a wide array of shoulder-related interventions.

Optimized dosing regimens: Ongoing studies are dedicated to determining the most effective and safe dosing regimens of dexmedetomidine for specific patient populations and surgical procedures. This includes investigations into the ideal concentration of dexmedetomidine, infusion rates, and bolus dosing strategies. The goal is to achieve the optimal balance between providing adequate sedation and analgesia while minimizing the risk of adverse effects. Tailoring dexmedetomidine dosing to the unique requirements of different patient profiles and surgical contexts is central to these efforts.

Patient-centered outcomes: The emphasis on patient-centered outcomes is gaining prominence in dexmedetomidine research. Studies are increasingly focusing on measuring outcomes that matter most to patients, such as overall satisfaction with their anesthesia experience, quality of postoperative recovery, and long-term shoulder function. Assessing the holistic impact of dexmedetomidine-enhanced blocks goes beyond pain relief and sedation, providing a more comprehensive understanding of how this technique affects the lives and well-being of patients over time.

Combination therapies: Research is actively exploring the potential synergy of dexmedetomidine with other agents, including non-opioid analgesics and adjuvants. The aim is to optimize pain control while minimizing opioid consumption further. By combining dexmedetomidine with complementary medications or techniques, researchers hope to create multimodal analgesic approaches that maximize the benefits of each component while reducing the reliance on opioids. This approach aligns with the broader goal of reducing opioid-related complications and improving postoperative recovery.

Comparison to alternative techniques: Emerging trends involve direct comparisons of dexmedetomidine-enhanced blocks with alternative regional anesthesia techniques used in shoulder surgery. This comparative research allows clinicians to make evidence-based decisions regarding the anesthesia approach. Dexmedetomidine's effectiveness and safety profile are assessed about other methods, such as continuous catheter-based infusions and intravenous opioid-sparing regimens. Comparative studies aim to provide a nuanced understanding of the advantages and limitations of each technique, facilitating informed clinical decision-making.

Areas Where Further Investigation is Needed

Long-term outcomes: The need for studies assessing long-term functional and pain outcomes remains paramount. While existing research has demonstrated the immediate benefits of dexmedetomidine-enhanced blocks, long-term follow-up is essential to understand how these interventions impact patients' quality of life, shoulder function, and pain relief beyond the immediate postoperative period. Longitudinal studies can provide insights into the durability of the analgesic effects and the potential for sustained improvements in patient outcomes. Comparing these long-term outcomes with patients who underwent traditional techniques will also help establish the enduring advantages of dexmedetomidine-enhanced blocks.

Patient selection criteria: Research efforts should focus on further refining patient selection criteria. Identifying which patients are most likely to benefit from dexmedetomidine-enhanced blocks is crucial for optimizing the use of this technique. Factors such as age, comorbidities, and the specific type of shoulder surgery should be considered when developing evidence-based guidelines for patient selection. Understanding the patient characteristics that predict the greatest benefit and safety from dexmedetomidine administration will help tailor its use to those who gain the most.

Safety and monitoring protocols: Continuous refinement of safety and monitoring protocols is imperative. As clinical experience with dexmedetomidine-enhanced blocks expands, there is an ongoing need to develop and enhance monitoring techniques that allow for the early detection and intervention of potential adverse effects. The optimization of safety protocols should involve real-time monitoring of vital signs, sedation levels, and adverse events to minimize complications and ensure patient safety. The development of standardized monitoring guidelines specific to this technique will further improve patient care.

Cost-effectiveness analysis: Evaluating the cost-effectiveness of dexmedetomidine-enhanced blocks is essential for healthcare institutions and policymakers. Studies should not only consider the direct costs of dexmedetomidine administration but also factor in the potential cost savings associated with reduced hospital stays, decreased opioid-related expenses, and accelerated recovery protocols. A comprehensive cost-effectiveness analysis will provide valuable insights into the economic implications of adopting this technique on a broader scale.

Comparative effectiveness: Comparative studies should be conducted to assess the effectiveness and safety of dexmedetomidine-enhanced blocks about other adjuvants or regional anesthesia techniques used in shoulder surgery. Comparative research can help clinicians make informed decisions about the choice of anesthesia technique by providing a comprehensive understanding of the benefits and limitations of each approach. Comparative effectiveness studies should encompass various surgical procedures and patient populations to yield robust and generalizable findings.

Potential Refinements or Innovations in Technique

Precision medicine: Precision medicine involves tailoring dexmedetomidine dosing and administration to individual patient characteristics. This includes genetics, age, weight, comorbidities, and pharmacogenomics. By leveraging genetic information, clinicians can optimize dosing regimens to maximize the effectiveness of dexmedetomidine while minimizing the risk of adverse effects. For example, genetic variations may influence the metabolism and response to dexmedetomidine, allowing for tailored treatment plans that enhance patient outcomes.

Advanced delivery systems: Innovative delivery systems like PCRA devices offer patients greater autonomy over their pain management. With PCRA, patients can self-administer dexmedetomidine on demand within predefined safety limits, allowing them to titrate their infusion postoperatively. This approach empowers patients and enhances their ability to manage pain effectively while minimizing the risk of over-sedation or excessive drug administration.

Pharmacokinetic modeling: Using pharmacokinetic modeling and simulation tools provides an opportunity to predict and individualize each patient's ideal dexmedetomidine dosing regimen. These models consider patient-specific factors such as age, weight, and surgical complexity, allowing for precise adjustments to optimize sedation and analgesia. Such modeling can help strike the delicate balance between achieving the desired clinical effects and preventing unwanted side effects, contributing to safer and more effective anesthesia.

Telemedicine and remote monitoring: Telemedicine and remote monitoring technologies can revolutionize postoperative care for patients receiving dexmedetomidine-enhanced blocks. Real-time data on patient comfort, vital signs, and side effects can be continuously transmitted to healthcare providers, enabling immediate intervention if complications or discomfort arise. Telemedicine also facilitates early recognition of adverse events, potentially reducing the severity and consequences of complications.

Enhanced safety algorithms: Developing advanced safety algorithms and decision-support systems is crucial for patient safety. These systems can integrate real-time data from patient monitoring devices, such as continuous blood pressure and heart rate monitoring, and use algorithms to identify trends or anomalies. Early warnings and alerts can be generated when potential complications are detected, prompting healthcare providers to intervene promptly. This proactive approach enhances patient safety by preventing or mitigating adverse events.

## Conclusions

In conclusion, incorporating dexmedetomidine into interscalene brachial plexus blocks for shoulder surgery significantly advances regional anesthesia. This comprehensive review has highlighted this approach's advantages, including extended analgesia, improved pain management, reduced opioid reliance, enhanced patient comfort, and preserved motor function. While acknowledging the need for meticulous patient selection, monitoring, and dosing considerations to mitigate potential risks, the utility of dexmedetomidine-enhanced blocks in clinical practice is undeniable. As we look to the future, ongoing research and collaboration among healthcare professionals are poised to refine techniques, optimize dosing strategies, and enhance patient outcomes. Dexmedetomidine has the potential to reshape the landscape of shoulder surgery anesthesia, ushering in a new era of improved pain control, patient satisfaction, and overall surgical experience.

## References

[1] (2023). Arthroscopic shoulder surgery for the treatment of rotator cuff tears. https://orthop.washington.edu/patient-care/articles/sports/arthroscopic-shoulder-surgery-for-the-treatment-of-rotator-cuff-tears.

[2] Cutts S, Prempeh M, Drew S (2009). Anterior shoulder dislocation. Ann R Coll Surg Engl.

[3] Maurya I, Garg R, Jain VK, Iyengar KP, Vaishya R (2021). Perioperative anaesthetic considerations for rotator cuff repair surgeries: A current concept review. J Clin Orthop Trauma.

[4] Hewson DW, Oldman M, Bedforth NM (2019). Regional anaesthesia for shoulder surgery. BJA Educ.

[5] Lanna M, Pastore A, Policastro C, Iacovazzo C (2012). Anesthesiological considerations in shoulder surgery. Transl Med UniSa.

[6] Basat HÇ, Uçar DH, Armangil M, Güçlü B, Demirtaş M (2016). Post operative pain management in shoulder surgery: suprascapular and axillary nerve block by arthroscope assisted catheter placement. Indian J Orthop.

[7] Lee BH, Qiao WP, McCracken S, Singleton MN, Goman M (2023). Regional anesthesia techniques for shoulder surgery in high-risk pulmonary patients. J Clin Med.

[8] Hwang JT, Jang JS, Lee JJ (2020). Dexmedetomidine combined with interscalene brachial plexus block has a synergistic effect on relieving postoperative pain after arthroscopic rotator cuff repair. Knee Surg Sports Traumatol Arthrosc.

[9] Luan H, Hao C, Li H, Zhang X, Zhao Z, Zhu P (2023). Effect of interscalene brachial plexus block with dexmedetomidine and ropivacaine on postoperative analgesia in patients undergoing arthroscopic shoulder surgery: a randomized controlled clinical trial. Trials.

[10] Polcaro L, Charlick M, Daly DT (2023). Anatomy, head and neck: brachial plexus. StatPearls [Internet].

[11] Basta M, Sanganeria T, Varacallo M (2023). Anatomy, shoulder and upper limb, suprascapular nerve. StatPearls [Internet].

[12] Lam JH, Bordoni B (2023). Anatomy, shoulder and upper limb, arm abductor muscles. StatPearls [Internet].

[13] Shetty P, Nayak SB, Kumar N, Thangarajan R, D'Souza MR (2014). Origin of medial and lateral pectoral nerves from the supraclavicular part of brachial plexus and its clinical importance - a case report. J Clin Diagn Res.

[14] Lung K, Lui F (2023). Anatomy, thorax, long thoracic nerve. StatPearls [Internet].

[15] Bayot ML, Nassereddin A, Varacallo M (2023). Anatomy, shoulder and upper limb, brachial plexus. StatPearls [Internet].

[16] Pester JM, Hendrix JM, Varacallo M (2023). Brachial plexus block techniques. StatPearls [Internet].

[17] Gertler R, Brown HC, Mitchell DH, Silvius EN (2001). Dexmedetomidine: a novel sedative-analgesic agent. Proc (Bayl Univ Med Cent).

[18] Giovannitti JA Jr, Thoms SM, Crawford JJ (2015). Alpha-2 adrenergic receptor agonists: a review of current clinical applications. Anesth Prog.

[19] Zhao Y, He J, Yu N, Jia C, Wang S (2020). Mechanisms of dexmedetomidine in neuropathic pain. Front Neurosci.

[20] Kaur M, Singh PM (2011). Current role of dexmedetomidine in clinical anesthesia and intensive care. Anesth Essays Res.

[21] Kaye AD, Chernobylsky DJ, Thakur P (2020). Dexmedetomidine in enhanced recovery after surgery (ERAS) protocols for postoperative pain. Curr Pain Headache Rep.

[22] Weerink MA, Struys MM, Hannivoort LN, Barends CR, Absalom AR, Colin P (2017). Clinical pharmacokinetics and pharmacodynamics of dexmedetomidine. Clin Pharmacokinet.

[23] Naaz S, Ozair E (2014). Dexmedetomidine in current anaesthesia practice- a review. J Clin Diagn Res.

[24] Wang Q, Chen C, Wang L (2022). Efficacy and safety of dexmedetomidine in maintaining hemodynamic stability in pediatric cardiac surgery: a systematic review and meta-analysis. J Pediatr (Rio J).

[25] Jin S, Liang DD, Chen C, Zhang M, Wang J (2017). Dexmedetomidine prevent postoperative nausea and vomiting on patients during general anesthesia: A PRISMA-compliant meta analysis of randomized controlled trials. Medicine (Baltimore).

[26] Zisquit J, Nedeff N (2023). Interscalene block. StatPearls [Internet].

[27] Singh S, Goyal R, Upadhyay KK, Sethi N, Sharma RM, Sharma A (2015). An evaluation of brachial plexus block using a nerve stimulator versus ultrasound guidance: a randomized controlled trial. J Anaesthesiol Clin Pharmacol.

[28] Krishna Prasad GV, Khanna S, Jaishree SV (2020). Review of adjuvants to local anesthetics in peripheral nerve blocks: current and future trends. Saudi J Anaesth.

[29] Guay J, Suresh S, Kopp S (2019). The use of ultrasound guidance for perioperative neuraxial and peripheral nerve blocks in children. Cochrane Database Syst Rev.

[30] Gasteiger L, Kirchmair L, Hoerner E, Stundner O, Hollmann MW (2023). Peripheral regional anesthesia using local anesthetics: old wine in new bottles?. J Clin Med.

[31] Kamel I, Ahmed MF, Sethi A (2022). Regional anesthesia for orthopedic procedures: What orthopedic surgeons need to know. World J Orthop.

[32] Brummett CM, Williams BA (2011). Additives to local anesthetics for peripheral nerve blockade. Int Anesthesiol Clin.

[33] Garimella V, Cellini C (2013). Postoperative pain control. Clin Colon Rectal Surg.

[34] Neal JM, Gerancher JC, Hebl JR, Ilfeld BM, McCartney CJ, Franco CD, Hogan QH (2009). Upper extremity regional anesthesia: essentials of our current understanding, 2008. Reg Anesth Pain Med.

[35] Malbrain ML, Langer T, Annane D (2020). Intravenous fluid therapy in the perioperative and critical care setting: executive summary of the International Fluid Academy (IFA). Ann Intensive Care.

[36] El-Boghdadly K, Pawa A, Chin KJ (2018). Local anesthetic systemic toxicity: current perspectives. Local Reg Anesth.

[37] Stafstrom CE, Carmant L (2015). Seizures and epilepsy: an overview for neuroscientists. Cold Spring Harb Perspect Med.

[38] Bao N, Shi K, Wu Y (2022). Dexmedetomidine prolongs the duration of local anesthetics when used as an adjuvant through both perineural and systemic mechanisms: a prospective randomized double-blinded trial. BMC Anesthesiol.

[39] Ping Y, Ye Q, Wang W, Ye P, You Z (2017). Dexmedetomidine as an adjuvant to local anesthetics in brachial plexus blocks: a meta-analysis of randomized controlled trials. Medicine (Baltimore).

[40] Imani F, Zaman B, De Negri P (2020). Postoperative pain management: role of dexmedetomidine as an adjuvant. Anesth Pain Med.

[41] Chen YK, Boden KA, Schreiber KL (2021). The role of regional anaesthesia and multimodal analgesia in the prevention of chronic postoperative pain: a narrative review. Anaesthesia.

[42] Kumar CM, Chua AW, Imani F, Sehat-Kashani S (2021). Practical considerations for dexmedetomidine sedation in adult cataract surgery under local/regional anesthesia: a narrative review. Anesth Pain Med.

[43] Chavan SG, Shinde GP, Adivarekar SP, Gujar SH, Mandhyan S (2016). Effects of dexmedetomidine on perioperative monitoring parameters and recovery in patients undergoing laparoscopic cholecystectomy. Anesth Essays Res.

[44] Li H, Liu J, Shi H (2021). Effect of dexmedetomidine on perioperative hemodynamics and myocardial protection in thoracoscopic-assisted thoracic surgery. Med Sci Monit.

[45] Abdallah FW, Dwyer T, Chan VW (2016). IV and perineural dexmedetomidine similarly prolong the duration of analgesia after interscalene brachial plexus block: a randomized, three-arm, triple-masked, placebo-controlled trial. Anesthesiology.

[46] Marhofer D, Kettner SC, Marhofer P, Pils S, Weber M, Zeitlinger M (2013). Dexmedetomidine as an adjuvant to ropivacaine prolongs peripheral nerve block: a volunteer study. Br J Anaesth.

[47] Chen BS, Peng H, Wu SN (2009). Dexmedetomidine, an alpha2-adrenergic agonist, inhibits neuronal delayed-rectifier potassium current and sodium current. Br J Anaesth.

[48] Ok HG, Baek SH, Baik SW, Kim HK, Shin SW, Kim KH (2013). Optimal dose of dexmedetomidine for sedation during spinal anesthesia. Korean J Anesthesiol.

[49] Zheng L, Zhao J, Zheng L, Jing S, Wang X (2020). Effect of dexmedetomidine on perioperative stress response and immune function in patients with tumors. Technol Cancer Res Treat.

[50] Kang R, Ko JS (2023). Recent updates on interscalene brachial plexus block for shoulder surgery. Anesth Pain Med (Seoul).

[51] Barreveld A, Witte J, Chahal H, Durieux ME, Strichartz G (2013). Preventive analgesia by local anesthetics: the reduction of postoperative pain by peripheral nerve blocks and intravenous drugs. Anesth Analg.

[52] Bohringer C, Astorga C, Liu H (2020). The benefits of opioid free anesthesia and the precautions necessary when employing it. Transl Perioper Pain Med.

[53] Schwenk ES, Mariano ER (2018). Designing the ideal perioperative pain management plan starts with multimodal analgesia. Korean J Anesthesiol.

[54] Vatsalya T, Waikar C, Singh M (2018). Comparison of intravenous bolus and infusion of dexmedetomidine on characteristics of subarachnoid block. Anesth Essays Res.

[55] Singh N, Gupta S, Kathuria S (2020). Dexmedetomidine vs dexamethasone as an adjuvant to 0.5% ropivacaine in ultrasound-guided supraclavicular brachial plexus block. J Anaesthesiol Clin Pharmacol.

[56] Chan VW (2003). Applying ultrasound imaging to interscalene brachial plexus block. Reg Anesth Pain Med.

[57] Gupta PK, Gupta K, Dwivedi AN, Jain M (2011). Potential role of ultrasound in anesthesia and intensive care. Anesth Essays Res.

[58] Li L, Zhao Y, Guo L, Lv X, Yu G (2020). Ultrasound guidance enhances the efficiency of brachial plexus block and ameliorates the vascular injury compared with nerve stimulator guidance in hand surgery patients. J Invest Surg.

[59] Yu SB (2012). Dexmedetomidine sedation in ICU. Korean J Anesthesiol.

[60] Jung HS, Seo KH, Kang JH, Jeong JY, Kim YS, Han NR (2018). Optimal dose of perineural dexmedetomidine for interscalene brachial plexus block to control postoperative pain in patients undergoing arthroscopic shoulder surgery: A prospective, double-blind, randomized controlled study. Medicine (Baltimore).

[61] Habibi V, Kiabi FH, Sharifi H (2018). The effect of dexmedetomidine on the acute pain after cardiothoracic surgeries: a systematic review. Braz J Cardiovasc Surg.

[62] Inagaki Y, Yamakage M, Sakamoto A, Okayama A, Oya N, Hiraoka T, Morita K (2022). The efficacy and safety of dexmedetomidine for sedation during surgery under epidural or spinal anesthesia: a randomized, double-blind, placebo-controlled study. Yonago Acta Med.

[63] Zhao X, Song Q, Wang Y, Zhang Q, Sun C (2023). Dexmedetomidine improves lung compliance in patients undergoing lateral decubitus position of shoulder arthroscopy: A randomized controlled trial. Medicine (Baltimore).

[64] Yang Q, Ren Y, Feng B, Weng X (2020). Pain relieving effect of dexmedetomidine in patients undergoing total knee or hip arthroplasty: A meta-analysis. Medicine (Baltimore).

[65] Goff J, Hina M, Malik N (2023). Can opioid-free anaesthesia be personalised? A narrative review. J Pers Med.

[66] Yoshitomi T, Kohjitani A, Maeda S, Higuchi H, Shimada M, Miyawaki T (2008). Dexmedetomidine enhances the local anesthetic action of lidocaine via an alpha-2A adrenoceptor. Anesth Analg.

[67] Kim N, Kim KH, Choi YS, Song SH, Choi SH (2022). Effect of dexmedetomidine on early postoperative cognitive function in patients undergoing arthroscopic shoulder surgery in beach chair position: a randomized double-blind study. J Clin Med.

[68] Eberl S, Preckel B, Bergman JJ, van Dieren S, Hollmann MW (2016). Satisfaction and safety using dexmedetomidine or propofol sedation during endoscopic oesophageal procedures: a randomised controlled trial. Eur J Anaesthesiol.

[69] Gao C, Huang T, Wu K (2022). Multimodal analgesia for accelerated rehabilitation after total knee arthroplasty: a randomized, double-blind, controlled trial on the effect of the co-application of local infiltration analgesia and femoral nerve block combined with dexmedetomidine. Brain Sci.

[70] Donatiello V, Alfieri A, Napolitano A (2022). Opioid sparing effect of intravenous dexmedetomidine in orthopaedic surgery: a retrospective analysis. J Anesth Analg Crit Care.

[71] Agarwal S, Aggarwal R, Gupta P (2014). Dexmedetomidine prolongs the effect of bupivacaine in supraclavicular brachial plexus block. J Anaesthesiol Clin Pharmacol.

[72] Kaye AD, Urman RD, Rappaport Y (2019). Multimodal analgesia as an essential part of enhanced recovery protocols in the ambulatory settings. J Anaesthesiol Clin Pharmacol.

[73] Garip L, Verbist J, Stragier H, Meyns J, Mesotten D, Vundelinckx J (2022). A comparative study of patient satisfaction about anesthesia with dexmedetomidine for ambulatory dental surgery. BMC Res Notes.

[74] Luo W, Xiong Y, Xiao ZY (2018). Clinical effects and safety evaluation of dexmedetomidine hydrochloride combined with etomidate fat emulsion in patients undergoing interventional treatment of stroke during anesthesia. Eur Rev Med Pharmacol Sci.

[75] Doo AR, Lee H, Baek SJ, Lee J (2021). Dexmedetomidine-induced hemodynamic instability in patients undergoing orthopedic upper limb surgery under brachial plexus block: a retrospective study. BMC Anesthesiol.

[76] Lee S (2019). Dexmedetomidine: present and future directions. Korean J Anesthesiol.

[77] Carlisle JB, Stevenson CA (2006). Drugs for preventing postoperative nausea and vomiting. Cochrane Database Syst Rev.

[78] Tobias JD, Leder M (2011). Procedural sedation: a review of sedative agents, monitoring, and management of complications. Saudi J Anaesth.

[79] Ludwig K, Sorrell M, Liu P (2009). Severe rash associated with dexmedetomidine use during mechanical ventilation. Pharmacotherapy.

[80] Boehm O, Baumgarten G, Hoeft A (2016). Preoperative patient assessment: identifying patients at high risk. Best Pract Res Clin Anaesthesiol.

[81] Kuhn C, Werdan K (2001). Hemodynamic monitoring. Surgical Treatment: Evidence-Based and Problem-Oriented.

[82] Aminiahidashti H, Shafiee S, Zamani Kiasari A, Sazgar M (2018). Applications of End-Tidal Carbon Dioxide (EtCO2) monitoring in emergency department; a narrative review. Emerg (Tehran).

[83] Dontukurthy S, Tobias JD (2021). Update on local anesthetic toxicity, prevention and treatment during regional anesthesia in infants and children. J Pediatr Pharmacol Ther.

[84] Ko KH, Jun IJ, Lee S, Lim Y, Yoo B, Kim KM (2015). Effective dose of dexmedetomidine to induce adequate sedation in elderly patients under spinal anesthesia. Korean J Anesthesiol.

[85] Elvir-Lazo OL, White PF, Yumul R, Cruz Eng H (2020). Management strategies for the treatment and prevention of postoperative/postdischarge nausea and vomiting: an updated review. F1000Res.

[86] Rodziewicz TL, Houseman B, Hipskind JE (2023). Medical error reduction and prevention. StatPearls [Internet].

[87] Janssen CF, Maiello *P, Wright MJ Jr, Kracinovsky KB, Newsome JT (2017). Comparison of atipamezole with yohimbine for antagonism of xylazine in mice anesthetized with ketamine and xylazine. J Am Assoc Lab Anim Sci.

[88] Haahr-Raunkjaer C, Mølgaard J, Elvekjaer M (2022). Continuous monitoring of vital sign abnormalities; association to clinical complications in 500 postoperative patients. Acta Anaesthesiol Scand.

